# High-frequency ultrasound detects biomechanical weakening in keratoconus with lower stiffness at higher grade

**DOI:** 10.1371/journal.pone.0271749

**Published:** 2022-07-20

**Authors:** Sunny Kwok, Xueliang Pan, William Liu, Andrew Hendershot, Jun Liu

**Affiliations:** 1 Department of Biomedical Engineering, The Ohio State University, Columbus, Ohio, United States of America; 2 Department of Biomedical Informatics, The Ohio State University, Columbus, Ohio, United States of America; 3 Department of Ophthalmology and Visual Sciences, The Ohio State University, Columbus, Ohio, United States of America; Nicolaus Copernicus University, POLAND

## Abstract

In vivo biomechanical characterization of the cornea remains a challenge. We have developed a high-frequency ultrasound elastography method, the ocular pulse elastography (OPE), to measure corneal axial displacement (CAD) induced by the ocular pulse. Here we compared CAD and a stiffness index derived from CAD between keratoconus patients and normal controls. We also explored the trend of these parameters with keratoconus grade. Twenty normal subjects and twenty keratoconus patients were recruited in this study. Corneal topography, tomography, intraocular pressure (IOP) and ocular pulse amplitude (OPA) were obtained in each measured eye. The cornea’s heartbeat-induced cyclic axial displacements were measured by high-frequency (50 MHz) ultrasound. A corneal stiffness index (CSI) was derived from CAD normalized against OPA. CAD and CSI were compared between normal and keratoconus groups, and across keratoconus grades. Keratoconus corneas had significantly greater CAD and lower CSI than normal controls (p’s<0.01). Both parameters correlated strongly with grade, in which CAD increased significantly (p = 0.002) and CSI decreased significantly (p = 0.011) with grade. These results suggested a biomechanical weakening in keratoconus which worsens at higher disease severity. This study also demonstrated the ability of high-frequency ultrasound elastography to provide a safe, quick, and accurate evaluation of the cornea’s biomechanical condition in vivo. The OPE-measured biomechanical metrics, when integrated with existing diagnostic criteria, may aid the decision-making in the early and definitive diagnosis and staging of keratoconus.

## Introduction

The cornea is the thin transparent tissue in the anterior ocular coat, responsible for 2/3 of the refractive power for focusing light onto the retina. About 90% of the cornea’s thickness is occupied by the stroma, which is composed of collagen fibers, proteoglycans, and sparsely distributed keratocytes to maintain and regulate the extracellular matrix [1]. The structure and the composition of the stroma largely determine the biomechanical properties of the cornea, which are critical for good vision. Keratoconus (KC) is a progressively degenerative corneal disease in which the cornea thins and protrudes into a conical shape, causing impaired vision [2, 3]. Current clinical diagnosis for KC is based on corneal topography and tomography to detect abnormalities in morphometric parameters such as thinnest pachymetry, maximal keratometry (K_max_), astigmatism, and maximum posterior elevation [4, 5]. Nonetheless, even with advanced structural imaging, it remains difficult to predict future KC progression and clinicians must still wait and monitor for evidence of manifestation of the disease.

Many previous studies have suggested a biomechanical alteration in KC corneas. Ex vivo mechanical testing of KC corneas showed a reduced modulus compared to normal corneas [6]. Microstructural imaging showed reduced collagen fiber interweaving [7] and smaller lamellar width [8], providing microstructural evidence of reduced mechanical strength. It was also shown that keratocyte apoptosis increased [9] and lysyl oxidase activity decreased [10] in KC, indicating tissue loss as well as mechanical weakening.

These observations suggest that the biomechanical properties of the cornea can potentially be a disease marker for KC. This has motivated the development of different techniques and methods to measure corneal biomechanics in vivo. The Ocular Response Analyzer (ORA, Reichert Technologies Inc., USA) and the Corvis ST (OCULUS, Germany) are two clinical devices that measure the whole cornea’s response to an air puff. Many studies have shown the potential of these methods to detect early-stage KC; however, air-puff induced corneal deformation is not only dependent on corneal properties, but also influenced by factors such as intraocular pressure (IOP) and scleral stiffness [11–13]. Imaging-based techniques using ultrasound [14–17], optical coherence tomography (OCT) [18–25], and Brillouin spectroscopy [26–28] have been proposed for noninvasive, spatially resolved measurements of corneal biomechanical properties. Each of these methods shows exciting potential, but major efforts are still needed before these techniques can be implemented in routine clinical practice to improve patient outcome.

We have previously developed and validated an ocular pulse elastography (OPE) technique which utilizes high-frequency ultrasound to measure in vivo corneal biomechanical response to the intrinsic ocular pulse in humans [15–17]. This technique utilizes an acoustic energy at the level of clinical ophthalmic ultrasound and can be an attractive option for quick and noninvasive clinical evaluation of corneal properties without external mechanical excitation. High-frequency ultrasound at 50 MHz offers both high spatial and high temporal resolution to measure ocular pulse induced corneal deformation, as shown in previous validation studies [16, 17]. The in vivo repeatability and trackability were also excellent in the presence of fixational eye motion [15]. In this study, we applied the OPE technique to characterize corneal axial displacement (CAD) and a biomechanical stiffness index derived from CAD in both KC patients and normal subjects. Correlations between these biomechanical parameters and clinical metrics for KC diagnosis such as thinnest pachymetry and K_max_ were explored.

## Materials and methods

### Participants

Twenty normal subjects with no known corneal pathology/surgeries or clinical signs of keratoconus and twenty keratoconus patients were enrolled in this study. Patients with a clinical diagnosis of keratoconus were recruited from those receiving eye care at the Ohio State University Havener Eye Institute. Keratoconus was identified by characteristic refractive and slit-lamp signs that include unstable refraction, oblique astigmatism, irregular retinoscopic and keratometry mires, and biomicroscopic signs such as Vogt striae, Fleischer ring, etc, and no history of corneal surgery in at least one eye. Patients with pellucid marginal degeneration in both eyes, undergoing current treatment for corneal disease in both eyes, and corneal pathology in both eyes were excluded. This study was conducted with written informed consent of all participants, in adherence to the tenets of the Declaration of Helsinki and with approval of The Ohio State University Institutional Review Board.

### Measurement setup and testing protocols

Each subject underwent three tests at the visit, in the order described below:

1. Corneal topography and tomography were first acquired in both eyes of the subject using Pentacam (Oculus, Wetzlar, Germany). Corneal curvature map, thickness map, K_max_, and thinnest pachymetry were recorded. Three repeated measurements were obtained in each eye. The Pentacam measurements were reviewed by a corneal specialist (AH) to determine the keratoconus grade (0 to 4 from mild to severe) using the Belin ABCD grading system [29].
2. Dynamic IOP over several cardiac cycles was measured using the PASCAL Dynamic Contour Tonometer (DCT; Ziemer USA, Alton, IL). Diastolic intraocular pressure (IOP) and ocular pulse amplitude (OPA) were recorded. Three repeated measurements were obtained in each eye.
3. Dynamic corneal motion was measured using the OPE technique [15, 16]. Four repeated measurements were recorded in each eye and the ultrasound images and signals were processed to obtain corneal axial displacement (CAD), corneal stiffness index (CSI), and central corneal thickness (CCT). Details of the OPE setup and measurements are described in our published study [15] and summarized below.

**Fig 1:**
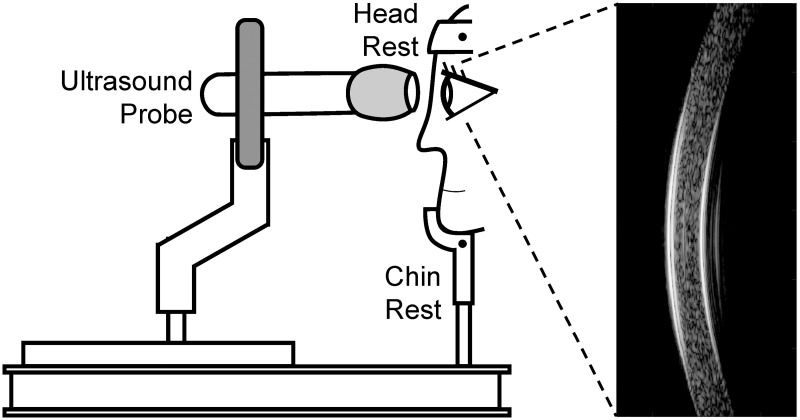
OPE measurement setup showing a subject sitting in front of an anti-vibration table with head secured into a chin-and-head rest mounted on the table. The ultrasound probe is mounted on a holder whose XYZ position can be adjusted by an operator. Ultrasound B-mode images and RF data of the cornea are collected along the nasal-temporal cross-section centered at the cornea apex.

Briefly, the subject sat with head placed on a head-and-chin rest mounted on an anti-vibration table (ScienceDesk Workstations, Thorlabs, Newton, NJ). A high-frequency ultrasound probe (50 MHz, MS700, Vevo 2100, FUJIFilm VisualSonics, Inc., Toronto, Canada) was secured to a probe-holder mounted on the table (Fig 1). The probe surface was covered with a cellular membrane and then a layer of eye lubricating gel (GenTeal Severe Dry Eye Relief, Alcon, Inc., Ft. Worth, TX). Anesthetic eye drops (Tetracaine HCl 0.5%, Bausch & Lomb, Bridgewater, NJ) were applied to both eyes prior to measurements.

For each measurement, the ultrasound probe was advanced towards the eye until the gel on the probe surface established contact with the cornea and the corneal image appeared on the monitor screen (Fig 1). Sound waves were transmitted through the gel layer into the cornea. One thousand consecutive B-mode frames and its radiofrequency (RF) data of the central 5.7 mm of the cornea were then acquired at 128 frames per second along the nasal-temporal axis. In normal eyes, all ultrasound scans were centered at the apex. For KC eyes, the ultrasound scans were also centered at the apex, except in corneas whose thinnest point was outside the pupil margin. For these corneas, two measurements were acquired at the apex and two more were collected at the cone region. Four repeated measurements were obtained in each eye, with additional gel applied to the probe surface as needed. The right eye (OD) was measured first, followed by the left eye (OS) in all subjects.

### Corneal displacements calculated from ultrasound speckle tracking

**Fig 2:**
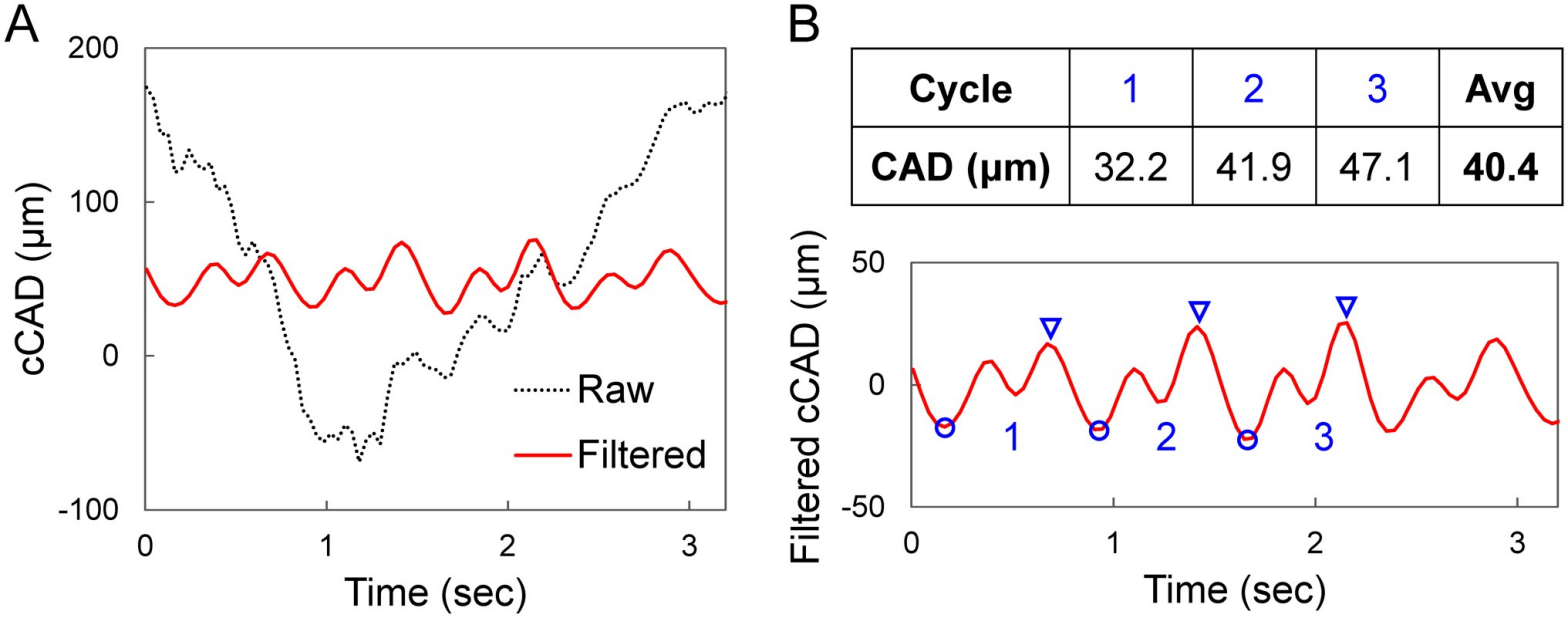
**A**. Raw and band-pass filtered cCAD curves from one OPE measurement in a KC patient showing eye motion in raw cCAD which was effectively removed by filtering. **B**. CAD for each measurement was calculated as the average trough (blue circle) to peak (blue triangles) distance for three automatically selected cycles.

Corneal displacements were calculated using an ultrasound speckle tracking algorithm described and validated previously [16, 17, 30, 31]. Briefly, the RF data were acquired and stored as 300 A-lines spaced at 19 μm intervals and each A-line was sampled at approximately 1.5 μm intervals (equivalent to a sampling frequency of 500 MHz). To perform speckle tracking on RF data, a region of interest (ROI) was defined in the reference frame (i.e., first acquired frame) of the RF data by automatic segmentation to generate an ROI approximately 4 mm in width bounded by the anterior and posterior surface of the cornea [15]. A dense rectangular grid was defined within the ROI, with about 200 grid points spaced by 13 × 10 pixels (axial × lateral), or approximately 19.5 μm × 190 μm. Kernels centered at each grid point containing 51 × 41 pixels were defined [16, 17]. To compute the displacement at each grid point, the unique speckle pattern within the kernel centered at the grid point was tracked in subsequent frames using cross-correlation. The maximum correlation coefficient value indicated the best match, and the corresponding kernel center was designated as the new location of the displaced grid point. Spline interpolation was used for subpixel tracking. To reduce processing time, we down-sampled the 1000 scanned frames by a factor of 5 and calculated displacements between down-sampled frames (200 in total). The displacement vector of each grid point was obtained between two consecutively sampled frames and accumulated over all 200 frames. Since the accuracy of axial displacement was much higher than that of lateral displacement due to higher spatial resolution and sampling density [17], only axial displacements were used for further analysis although lateral displacements were also calculated. The average axial displacement of all grid points within the ROI was plotted as the cumulative corneal axial displacement (cCAD) curve (Fig 2A).

The cCAD signal was filtered to remove noise using a band-pass filter described in our previous work [15]. Briefly, the heart rate frequency (F_HR_) for each measurement was first identified automatically within the frequency spectrum. Frequency components below 0.9F_HR_, above 3F_HR_, and those with magnitudes below 10% of the maximum peak were removed. CAD was calculated as the average trough-to-peak magnitudes of three displacement cycles that were automatically identified (Fig 2B) based on similarity of cycle width and height. Troughs were first identified as local minima, and the corresponding peak was identified as the maxima between two consecutive troughs no less than 1/5 of the heart rate distance from the first trough. Four repeated measurements (12 trough-to-peak amplitudes in total) were used to obtain the average CAD of each eye.

Since the CAD is a response of the cornea to the internal OPA at each heartbeat, we define the corneal stiffness index as follows:

$$\text{Corneal}\;\text{Stiffness}\;\text{Index}\;\left(\text{CSI}\right)=\text{OPA}/\text{CAD}$$ (1)

### Central corneal thickness measured by high-frequency ultrasound

An automated algorithm [15] was developed to calculated CCT from the scanned ultrasound images. The algorithm identified the anterior and posterior cornea surfaces based on RF signal intensity in the 500^th^ B-mode frame, which was the mid-point of data acquisition during each measurement. MatLab (The Math Works, Inc., Version 2020a) functions *findpeaks* (Signal Processing Toolbox) and *edge* (Image Processing Toolbox) were applied to the RF data following a similar procedure for identifying ROI. The smallest corneal thickness from all scanned A-lines on this frame was taken from each measurement and the average was recorded as the CCT.

### Receiver operating characteristic (ROC) curves

Receiver operating characteristic (ROC) curves were plotted to evaluate the predictive accuracy of OPE-measured corneal parameters (CAD and CSI) in comparison to clinical metrics (K_max_ and thinnest pachymetry). The curves were obtained by plotting sensitivity (true positive rate) vs 1 –specificity (false positive rate). Area under the curve (AUC) was calculated for each ROC curve using the MatLab internal function *trapz*. Diagnostic power was considered excellent for AUC values between 0.9–1, good for AUC values between 0.8–0.9, fair for AUC values between 0.7–0.8, poor for AUC values between 0.6–0.7 and failed for AUC values between 0.5–0.6 [32].

### Statistical analysis

The primary objective of this study was to measure CAD and CSI in normal and KC eyes, and to test if they were significantly different between the two groups. A sample size of 20 subjects per group provided at least 80% power to detect an effect size of 1.0 (Cohen’s d = 1.0) between normal and KC eyes. The effect size of 1 was chosen based on the modulus difference between these two groups reported by Andreassen et al. [6].

Summary statistics (mean and standard deviations) were generated for IOP, OPA, CAD, CSI, K_max_, thinnest pachymetry and CCT in normal and keratoconus eyes were used to generate the summary statistics (mean and standard deviations). To compare between groups, linear mixed models for repeated measures were used to account for the association between paired eyes of the same subject where group was the fixed effect and subject was the random effect. Sensitivity analysis after adjusting for age and IOP as covariates were also conducted to confirm if the conclusions of the differences were robust to model selection. Pearson correlations were used to summarize the associations of the measures between OD and OS by different groups. Association of CAD/CSI and keratoconus grade were also evaluated using linear mixed models for repeated measures to account for association between paired eyes of the same subject where grade (KC grade ranged from 0 to 4 and the normal grade was assigned as -1) was set as fixed effect and subject as random effect. The ROC curves were verified from logistic regression where group (reference is the normal group) was the response variable and the corresponding parameter of interest was explanatory variable. The associations between CAD/CSI and K_max_/thinnest pachymetry/IOP were also explored using scatter plots. All statistical analyses were conducted using SAS (version 9.4, SAS Inst Inc, NC, USA). A p-value ≤ 0.05 is considered as statistically significant.

## Results

**Fig 3:**
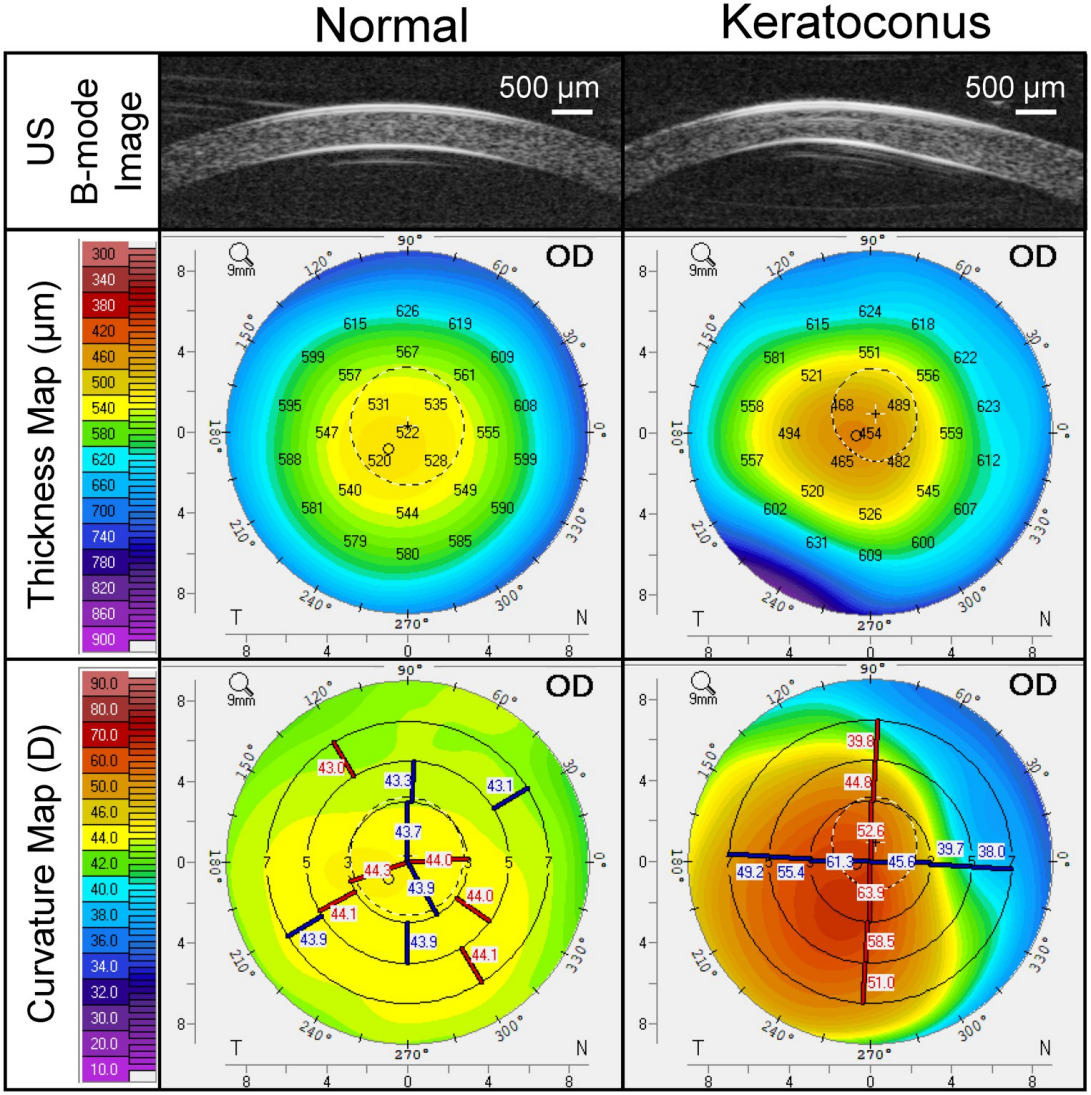
High-frequency ultrasound B-mode images of the central 5.7 mm of the cornea from a normal subject (left) and a grade-4 KC patient (right), along with their corresponding thickness and curvature maps obtained by Pentacam.

The normal group had an age range of 22 to 69 years old (43±15 years old). Pentacam readings confirmed the absence of abnormal topography in these subjects. A total of 40 normal eyes were included in this study. The KC group had an age range of 18 to 64 years old (37±12 years old), and the thinnest pachymetry was between 271 to 545 μm in this group. Five eyes from five KC patients were excluded due to scarring and/or other corneal pathologies. A total of 35 KC eyes were included in this study. B-mode ultrasound images and Pentacam thickness and curvature maps from a normal subject and a KC patient are shown in Fig 3. Pentacam, DCT and OPE measurements, and comparisons between normal and KC groups are summarized in Table 1. In one of the normal subjects, DCT measurements (IOP and OPA) were not available in both eyes.

**Table 1:** Summary statistics for age, IOP, OPA, K_max_, thinnest pachymetry, CCT, CAD, and CSI in 20 normal subjects (40 eyes) and 20 keratoconus patients (35 eyes).

	Normal	KC	P*	P**
Age (years)	43 ± 15	37 ± 12	0.20	NA
Diastolic IOP (mmHg)	16.3 ± 2.5	15.6 ± 3.4	0.47	NA
OPA (mmHg)	2.56 ± 0.84	2.24 ± 0.89	0.21	0.26
K_max_ (D)	45.0 ± 1.91	57.4 ± 13.4	**<0.001**	**<0.001**
Thinnest Pachymetry (μm, Pentacam)	531 ± 28	447 ± 63	**<0.001**	**<0.001**
CCT (μm, Ultrasound)	535 ± 30	448 ± 74	**<0.001**	**<0.001**
CAD (μm)	34.6 ± 10.3	47.8 ± 16.5	**0.004**	**0.009**
CSI (mmHg/μm)	0.082 ± 0.038	0.051 ± 0.022	**0.003**	**0.007**

* Without IOP and age as covariates

** IOP and age as covariates in the linear mixed model

**Fig 4:**
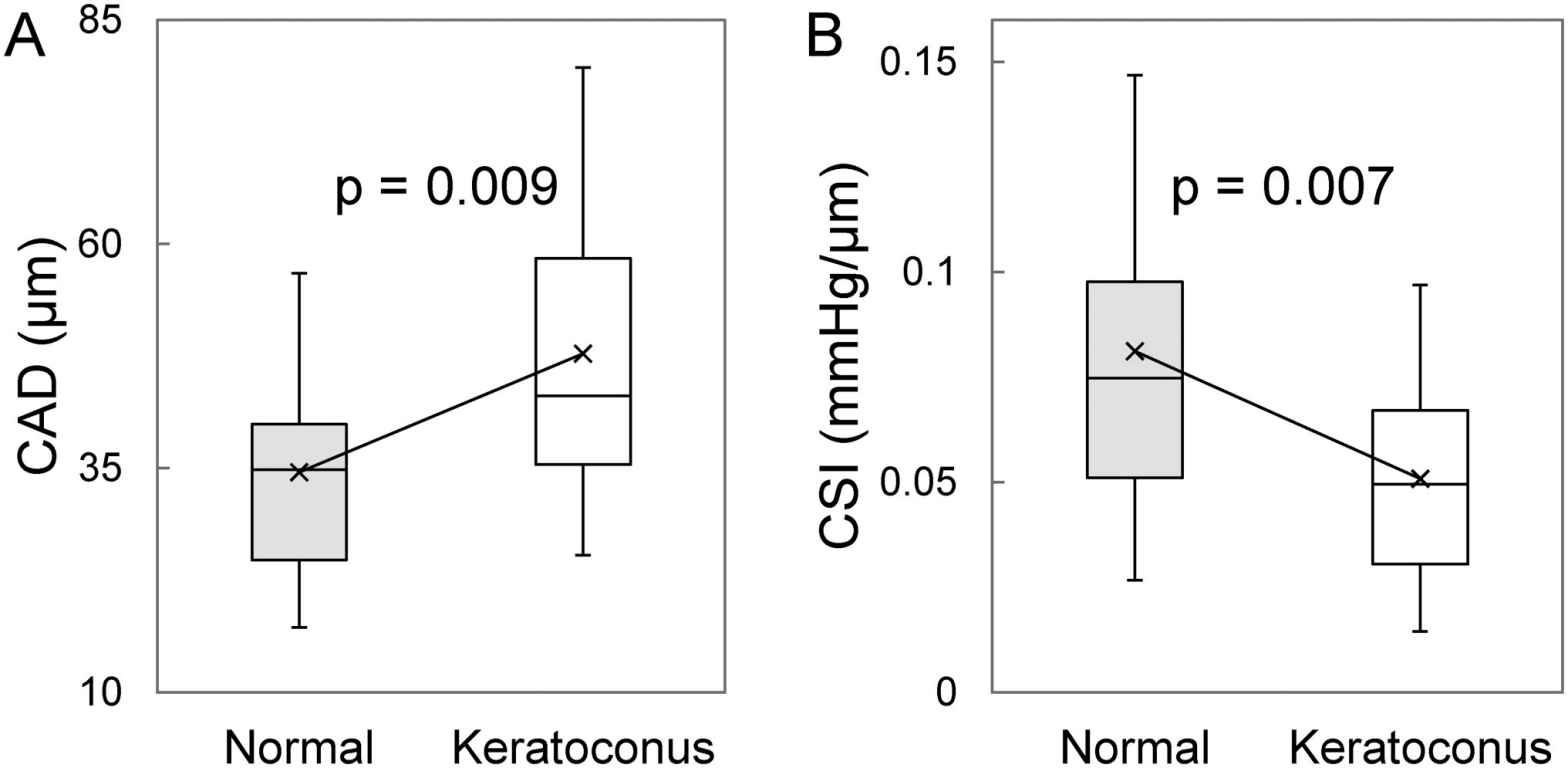
Boxplots showing the comparison of **A**. corneal axial displacement (CAD) and **B**. corneal stiffness index (CSI) in normal and keratoconus subjects. Average values for each group are marked with ×.

**Fig 5:**
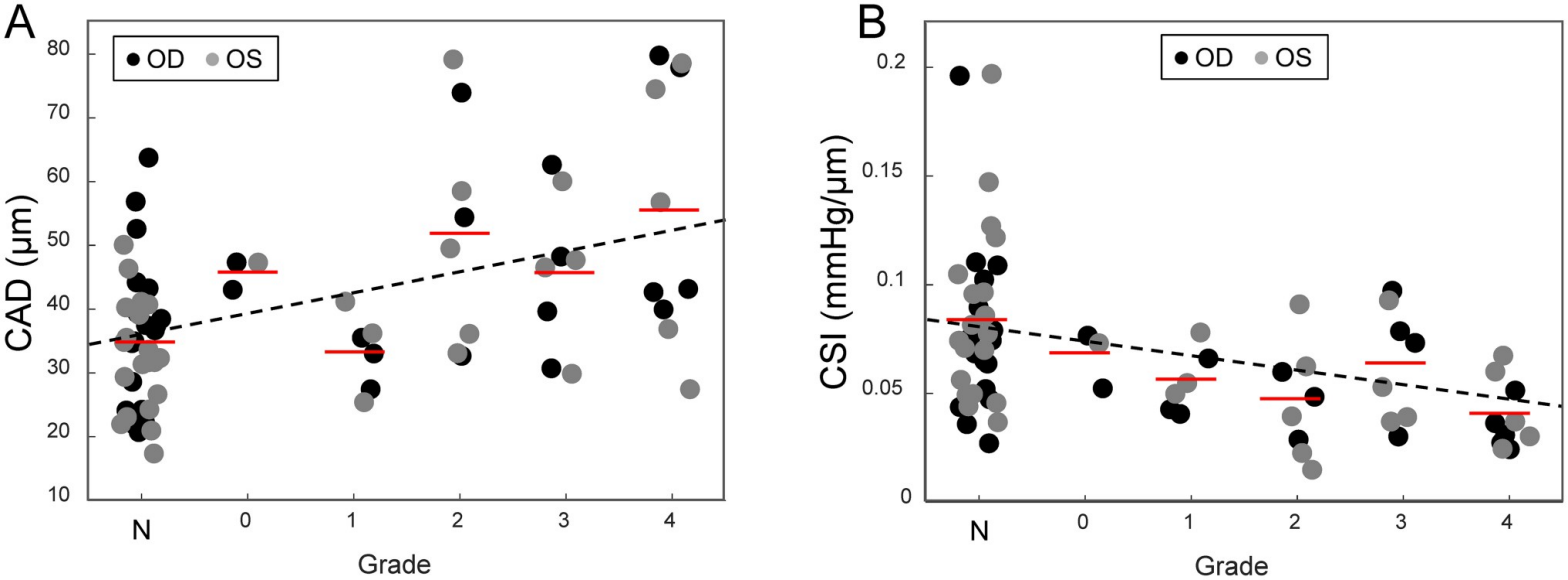
Correlation of CAD and CSI to KC grade (N: normal). **A**. CAD increased significantly at higher grade (p = 0.002). **B**. CSI decreased significantly at higher grade (p = 0.011). The red lines mark the average value within each grade.

Age, IOP and OPA were not different between the normal and the KC group. CCT and thinnest pachymetry were significantly lower in KC than normal (CCT: 461±97 μm vs 535±30 μm, p<0.001; thinnest: 447±63 μm vs 531±28 μm, p<0.001). In the 35 KC eyes, 14 had the thinnest point outside the pupil margin as indicated by the dashed circle in Pentacam images. There was no significant difference in CAD (p = 0.38, n = 14) between apex (39.5±12.1 μm) and cone (37.3±9.7 μm) in these patients. Therefore, the apex and cone CAD measurements were combined in the following analysis. CAD was significantly higher in KC than normal (47.8±16.5 μm vs 34.6±10.3 μm, p = 0.004; Table 1, Fig 4A). CSI was significantly lower in KC than normal (0.051±0.022 mmHg/μm vs 0.081±0.038 mmHg/μm, p = 0.003; Table 1, Fig 4B). Sensitivity analysis considering age and IOP as covariates yielded the same conclusions with slightly changed P values (Table 1). Grading according to the Belin ABCD system resulted in three eyes in grade 0, six in grade 1, eight in grade 2, eight in grade 3, and ten in grade 4. The mean CAD for each grade was 45.8±2.45 μm, 33.0±5.87 μm, 52.1±17.9 μm, 45.6±12.0 μm, and 55.7±20.2 μm, respectively. Mean CAD was found to significantly increase with higher KC grade (p = 0.002, Fig 5A). Mean CSI for each grade was 0.067±0.013 mmHg/μm, 0.055±0.014 mmHg/μm, 0.046±0.014 mmHg/μm, 0.062±0.026 mmHg/μm, and 0.039±0.015 mmHg/μm, respectively. Mean CSI significantly decreased at high KC grade (p = 0.011, Fig 5B).

It is noted that we reported an average CAD of about 47.1 μm in normal cornea in a previous study [15], which was higher than the average of 34.6 μm measured in the present study. This difference is caused to a change we made in the bandpass frequency applied to filter the cCAD curves. In order to remove apparent low-frequency eye motion noise, we further optimized the filter by raising the lower threshold of the bandpass filter while preserving frequencies of the first three harmonics of the subject’s heart rate. This change resulted in a decrease of CAD measured from both normal and KC eyes.

**Fig 6:**
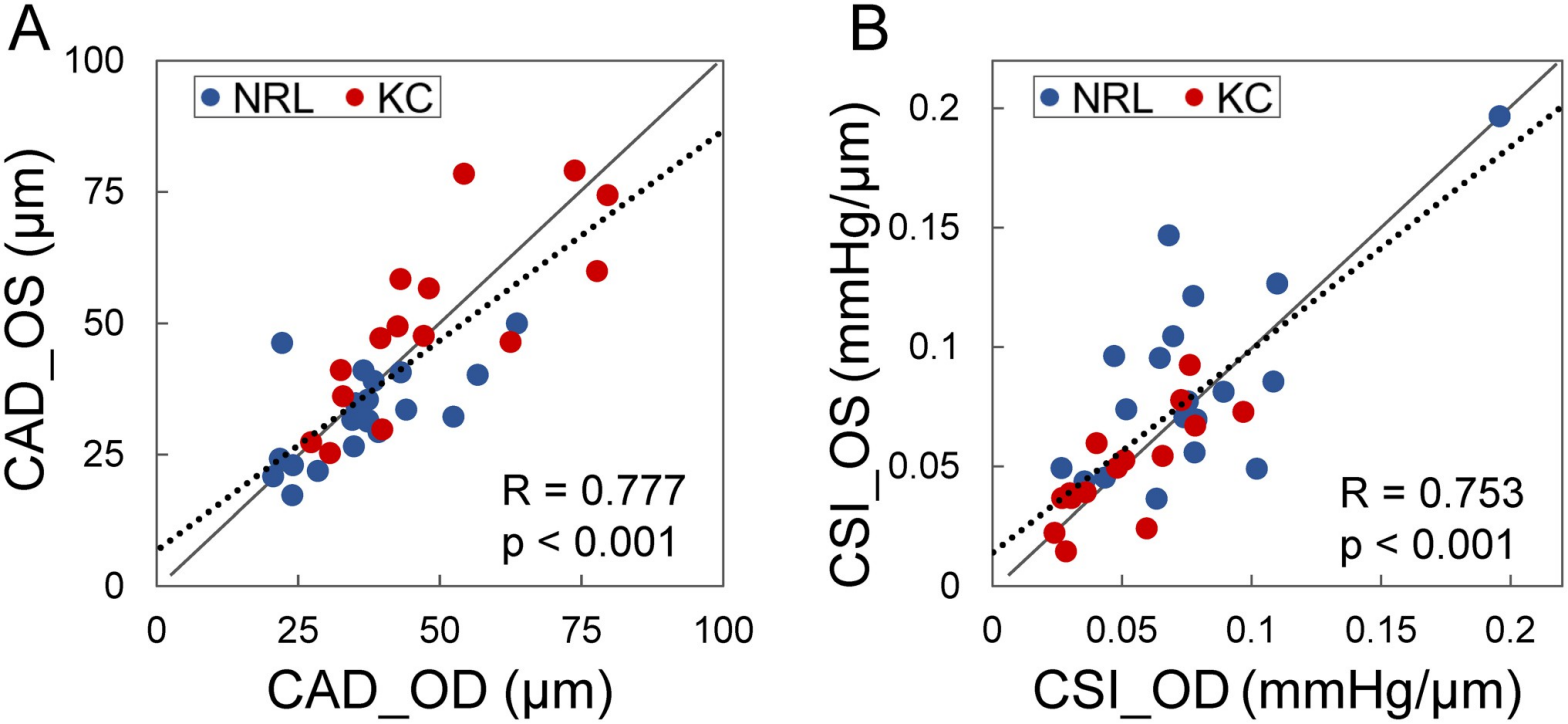
Both CAD (A) and CSI (B) show strong bilateral symmetry in both normal and KC eyes.

**Fig 7:**
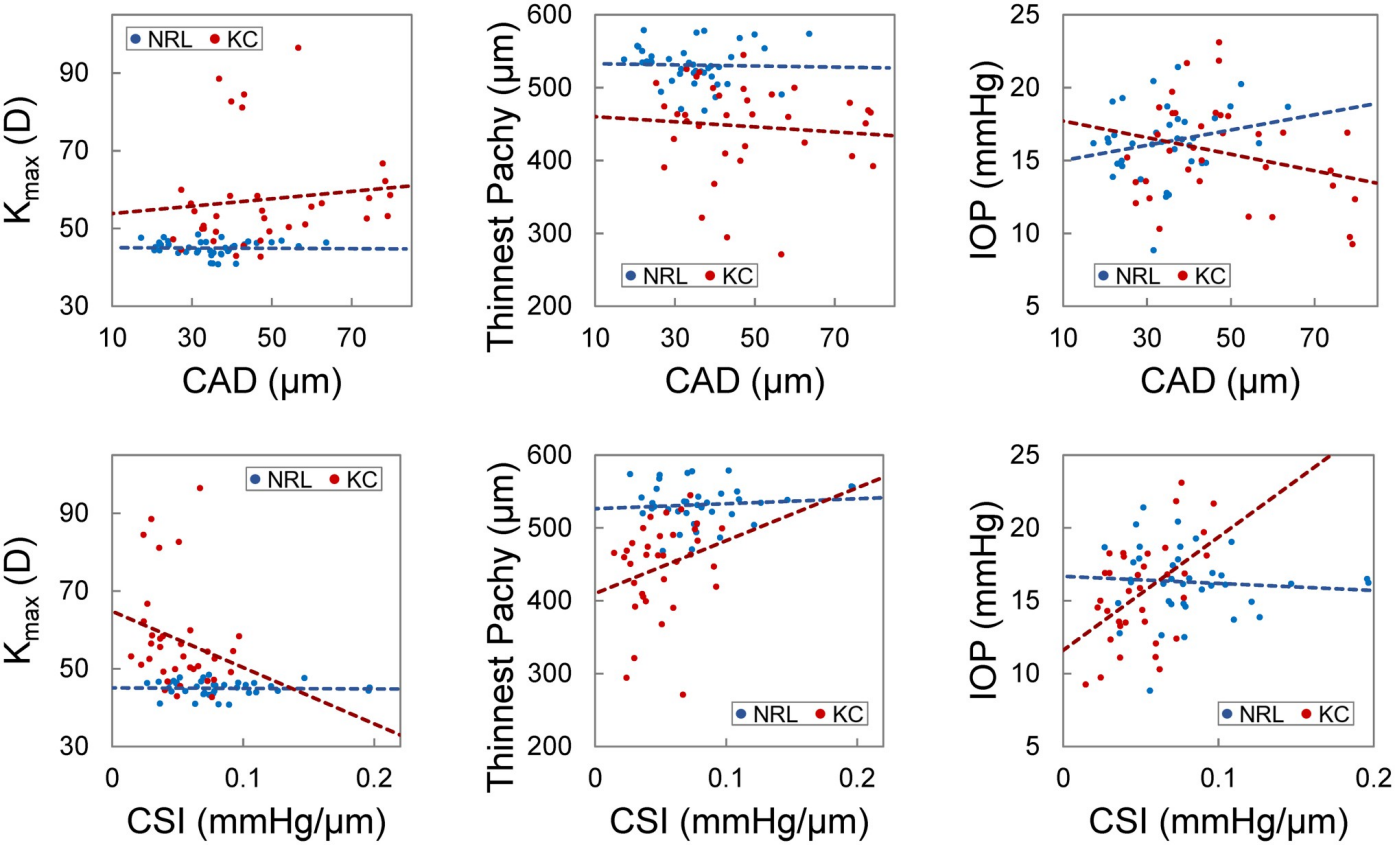
Scatter plots of CAD (top), CSI (bottom) vs. K_max_, thinnest pachymetry, and IOP. These plots indicated potential trends of correlation between CSI and K_max_ or thinnest pachymetry and a significant correlation between CSI and IOP (p = 0.003) in KC eyes (red dots) but not in normal controls (blue dots). CAD was not correlated with K_max_, thinnest pachymetry, or IOP in either group.

CAD and CSI showed a strong correlation between the left and right eye (R = 0.78 and 0.75, respectively, p’s<0.001, Fig 6). When the normal group and the KC group were examined separately, the bilateral symmetry remained strong (CAD: R = 0.63 and 0.79; CSI: R = 0.68 and 0.78). CCT of the left and right eyes was strongly correlated in the normal group (R = 0.99, p<0.001), but not in the KC group (R = 0.13, p = 0.65). CAD and CSI were not correlated with age, K_max_, thinnest pachymetry, or IOP in the normal group. CAD was not correlated with these parameters in the KC group either. Scatter plots indicated potential trends between CSI and K_max_/thinnest pachymetry in the KC group (Fig 7). There was a positive correlation (R = 0.50, p = 0.003) between CSI and IOP in the KC group.

**Fig 8:**
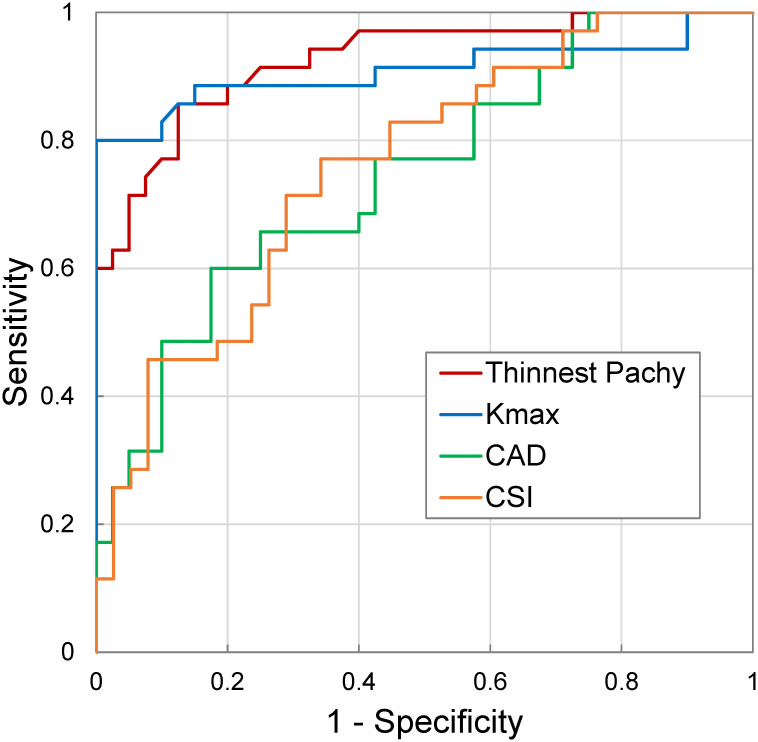
ROC curves of the OPE-measured CAD and CSI, as well as thinnest pachymetry and K_max_ in all measured subjects. Thinnest pachymetry and K_max_ had an AUC greater than 0.9, while CAD and CSI had an AUC between 0.7–0.8.

ROC curves for thinnest pachymetry, K_max_, CAD, and CSI are shown in Fig 8. Thinnest pachymetry had the highest AUC (0.93), followed by K_max_ (0.91), CSI (0.76) and CAD (0.75).

## Discussion

In this study, we used the OPE technique to quantify corneal displacement in response to the intrinsic ocular pulse in normal and KC eyes. The very high-frequency ultrasound used in the OPE technique acquires cross-sectional corneal images at a high resolution and a high frame rate, capturing cyclic corneal motion in response to the heartbeat. Corneal biomechanical metrics such as CAD and CSI were computed from analyzing the RF signals across several heartbeats. Major findings are: 1. KC corneas had significantly higher CAD and lower CSI as compared to normal corneas, consistent with previous findings of corneal mechanical weakening in KC; 2. CAD was positively correlated with KC grade, suggesting progressive weakening at greater disease severity; and 3. CSI decreased at higher grade, again, suggesting lower corneal stiffness in higher grade KC.

Previous uniaxial tensile tests identified significantly lower mechanical strength in KC corneas with a reduced tensile modulus of almost 50% [6]. High-speed Scheimpflug imaging demonstrated reduced resistance to deformation induced by air puff in KC eyes in vivo [33, 34]. Optical coherence elastography (OCE) indicated the ratio of anterior to posterior displacement in response to an applanation force was altered in KC [24]. Brillouin imaging has shown evidence of reduced longitudinal modulus derived from Brillouin frequency shift in KC eyes [27, 28]. Our study showed an increased CAD in KC eyes. The cornea’s outward movement in response to the ocular pulse, quantified by CAD in the current study, was on average ~1.4 times greater in KC than normal eyes. No difference was found between the OPA in these two groups. Thus, it stands to reason that the larger displacement suggests a more compliant cornea in KC eyes. This is further supported by the reduced CSI in KC, which is essentially CAD normalized by OPA. CSI in normal corneas was ~1.6 times higher on average compared to KC. These results are consistent with previous findings and further support the idea of a compromised mechanical stiffness in KC corneas.

Furthermore, we detected significant trends for both CAD and CSI in relation to KC grade. CAD increased and CSI decreased at higher grade. Both of these trends support the biomechanical decompensation hypothesis [35] that postulates a progressive mechanical weakening of the corneal stroma as KC progresses. These results also suggest that biomechanical metrics such as CAD or CSI may add diagnostic value to predict KC development and progression. This is of particular interest because we now have an effective treatment, i.e., corneal crosslinking, to stop progressive disease [36]. Physicians and patients will be more convinced to undertake the crosslinking treatment before apparent disease progression if more definitive evidence of high progression risk such as a low CSI is available. Alternatively, it would be safer to closely monitor for progression if biomechanical metrics do not deviate significantly from normal. Future studies are needed to investigate the predictive value of OPE-measured biomechanical metrics for KC progression.

We have previously reported a high bilateral symmetry of CAD in normal eyes [15]. In this study, strong bilateral symmetry of CAD and CSI was observed in both normal and KC eyes (Fig 6). While the symmetry was expected in normal eyes, it is interesting that it persisted in KC eyes despite asymmetry in grade. Four out of the twenty KC patients had the same KC grades in both eyes, while eleven had different grades between their two eyes. The other five patients only had one eye measured due to exclusion criteria. Although very rarely did we encounter cases in which the paired KC eyes were more than two grades apart, the bilateral symmetry in KC eyes suggested that systematic factors such as genetics may be a dominant determinant of the baseline biomechanical properties of the cornea [37]. Future studies are needed to understand how innate and environmental factors contribute to corneal biomechanical properties.

We explored the correlations between OPE-derived corneal biomechanical metrics (i.e., CAD and CSI) and morphometric parameters K_max_ and thinnest pachymetry. In general, there was no correlation between the biomechanical and morphometric parameters in the normal group, suggesting independence between these parameters, which is expected. There were some interesting potential trends in the KC group between CSI and K_max_ or thinnest pachymetry (Fig 7). Lower CSI (weaker cornea) appeared to be associated with higher K_max_ and smaller thinnest pachymetry, all indicators of more severe KC. Seiler et al. reported a significant correlation between Brillouin frequency shift at the maximum posterior elevation point and KC-metrics such as K_max_ and thinnest pachymetry [27]. Given that almost no trends were observed in normal corneas, the association between biomechanical and morphometric parameters in KC is likely due to progressive change in these parameters during KC progression.

**Fig 9:**
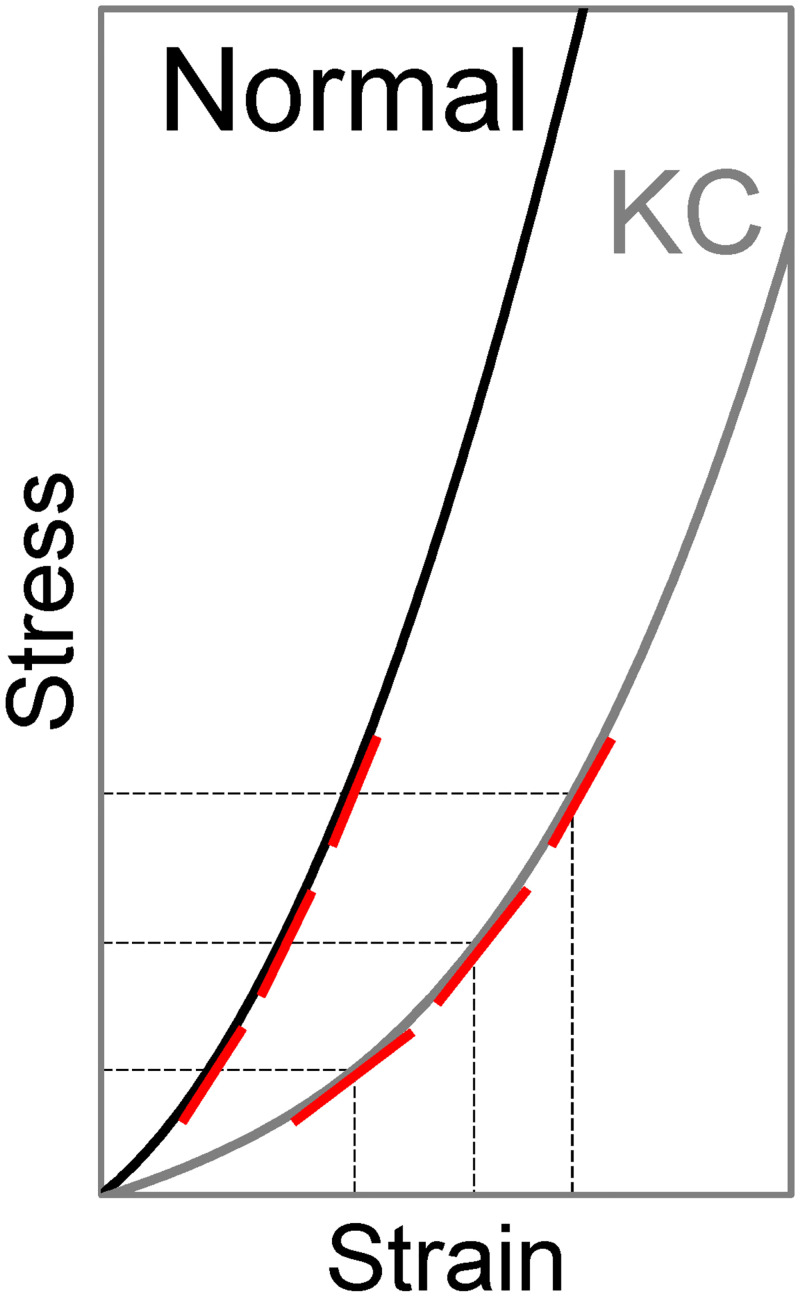
Comparison of stress-strain relationships in normal and KC corneas. Within similar levels of stress induced by physiological IOP, KC corneas have lower stiffness (smaller slopes, indicated by red lines) but experiences more drastic changes in stiffness when stress/IOP changes. Normal corneas have higher stiffness but smaller changes in stiffness when stress changes.

We also explored the correlation between CAD/CSI and IOP. CAD was not correlated with IOP in either KC or normal groups. There was an interesting positive correlation between CSI and IOP in the KC group but not in the normal group. IOP was measured by dynamic contour tonometry (DCT) in the present study, which has less or no dependance on corneal properties or thickness [38]. Therefore, potential tonometry errors likely cannot explain the association between CSI and IOP in KC eyes. We propose a possible explanation based on the biomechanical behavior of the cornea. As shown in Fig 9, KC is weaker than normal and therefore its stress-strain curve is shifted to the right as compared to normal. Within the IOP range seen in the present study, the normal corneas have a higher stiffness (larger slopes of the stress-strain curve), but the slopes change minimally within the stress levels corresponding to the normal IOP. Within the same stress range, KC corneas have a lower stiffness (smaller slopes) but experience significant stress-related stiffening (larger slope at higher IOP) resulting in a detectable correlation between IOP and stiffness (i.e., CSI measured in this study). Further studies are needed to explore this possibility and its potential application in detecting keratoconus.

Although CAD and CSI were significantly different between normal and KC corneas, there was substantial variance within each group and the difference between group was at the level of the standard deviation within group. This explains the lower AUC for CAD and CSI compared to K_max_ or thinnest pachymetry (Fig 8), suggesting that CAD or CSI alone does not have sufficient sensitivity and specificity for KC diagnosis. Interestingly, this result was remarkably similar to what Seiler et al. found in their study, showing an AUC between 0.7–0.8 for biomechanical metrics, and an AUC>0.9 for K_max_ and thinnest pachymetry [27]. Three aspects are noted to interpret this result. First, in both the Seiler study and our study, only adults (age 18 and above) were included. KC commonly starts in puberty and progresses more rapidly in children than in adults. CAD/CSI in this population has not been obtained and it will be interesting to re-evaluate AUC in this population or after including these younger patients. Second, a biomechanical metric can be combined with other diagnostic metrics to further improve sensitivity and specificity. Future studies are needed to design diagnostic criteria that optimally integrate biomechanical metrics into the existing clinical criteria to improve early detection and overall diagnostic effectiveness. Third, as discussed earlier, our results suggest that the biomechanical metrics will likely provide independent and additional information regarding progression risk. For cases with borderline topography and tomography, a biomechanical evaluation can better inform physicians to recommend or decide a treatment or follow up plan to avoid sight-threatening complications such as corneal ectasia.

The results of this study should be considered with a few limitations in mind. First, the OPE technique requires indirect contact between the ultrasound probe and the subject’s cornea, which is less convenient than noncontact optical methods. Measures were taken to minimize patient discomfort during examination by applying anesthetic eye drops in both eyes and an ultrasound-conducting, eye-lubricating gel in the measured eye. None of the participants we measured reported any pain or irritation during or after OPE measurements, suggesting it is tolerated well by both normal and KC patients. It is noted that each single OPE measurement takes about 30 seconds to complete, after the patient’s head is situated at the chin-and-head rest. Second, the sample size of each KC grade was small, especially for mild KC (grade 0 and 1). Although significant trends associating CAD and CSI changes with KC grade was identified, this result needs further validation in a larger sample size. We also plan to evaluate the association between biomechanical metrics and topo/tomographic metrics in a larger sample size. Third, the current study only analyzed the averaged CAD and CSI over the measured corneal cross-section. Our method is capable of mapping and resolving regional responses with adequate spatial resolution. Based on our current results in this study, CAD does not vary significantly spatially, which is expected as very small strains (i.e., spatial gradients of displacements) are produced by the ocular pulse. Nonetheless, the spatial variance of corneal strains or other derived parameters may better reflect the intrinsic biomechanical properties of the cornea and thus warrant future investigation to better detect the altered responses of the cone, improving early detection. Lastly, although CSI is a quantitative stiffness measure, it is different from modulus. For a tissue like the cornea that is nonlinear, anisotropic, and heterogenous, it is a challenging task to identity a material property that can adequately characterize such complex behavior and is also clinically useful. Future studies are needed to take advantage of the displacement data and in vivo cross-sectional images generated by OPE to develop patient-specific computational models in combination with inverse finite element analysis to derive tissue material properties for greater diagnostic value.

In summary, this study demonstrated the potential clinical application of the OPE technique for rapid and noninvasive corneal biomechanical evaluation using high-frequency ultrasound. OPE-measured CAD and CSI may become new biomechanical metrics for the detection and characterization of KC progression. Combined with other clinical parameters, the OPE method may contribute to KC diagnosis and aid the management of ecstatic corneal diseases.

## Supporting information

S1 Data (XLSX)
